# Young patient with hantavirus-induced myocarditis detected by comprehensive cardiac magnetic resonance assessment

**DOI:** 10.1186/s12879-018-3658-8

**Published:** 2019-01-06

**Authors:** Patrick Krumm, Tanja Zitzelsberger, Meinrad Gawaz, Simon Greulich

**Affiliations:** 1 Department of Radiology, University of Tübingen, Tübingen, Germany; 2 Department of Cardiology and Cardiovascular Diseases, University of Tübingen, Otfried-Müller-Strasse 10, 72076 Tübingen, Germany

**Keywords:** Hantavirus, Myocarditis, Cardiac magnetic resonance

## Abstract

**Background:**

We report a case of hantavirus-induced myocarditis in a young adult. Hantavirus showed a rapid increase of infections in the year 2017. Only scarce data is available about potential myocardial involvement in hantavirus infections. With ECG and echocardiography providing often inconclusive results, a multiparametric cardiac magnetic resonance protocol with distinct myocardial tissue characterization seems to be the adequate tool for detecting even slight myocardial alterations.

**Case presentation:**

This case started with the presentation of young adult suffering from headache and abdominal pain. Thrombocytes were decreased, creatinine was elevated, and there was massive proteinuria. Puumala virus IgG ELISA turned out to be positive, and specific antibodies (IgG and IgM) could be detected in the serum, and confirmed by immunoassay. The patient was admitted to the nephrology department for supportive therapy. Few days later, the patient reported chest pain and dyspnea. High sensitivity troponin I rose up to 0.32 μg/l (normal range below 0.04 μg/l) with an increase of the creatinkinase to 319 U/l (normal max. 190 U/l), no dynamic ECG changes could be observed. Echocardiography revealed a normal left ventricular function without regional wall motion abnormalities, no pericardial effusion or valve abnormalities, coronary artery disease could be excluded by computed tomography. A multiparametric cardiac magnetic resonance protocol including recent mapping techniques confirmed myocardial involvement induced by acute hantavirus infection. In the next few weeks, the patient’s state of health rapidly improved and symptoms of chest pain and dyspnea disappeared. Follow up multiparametric CMR exam showed substantial decrease of the previously observed myocardial alterations during acute hantavirus infection suggesting myocardial healing.

**Conclusions:**

This case demonstrates that a CMR protocol including recent mapping techniques and established late gadolinium enhancement technique is an adequate non-invasive tool for both 1) initial detection, and 2) follow up of patients with hantavirus-induced myocarditis, which might be more common than previously known.

**Electronic supplementary material:**

The online version of this article (10.1186/s12879-018-3658-8) contains supplementary material, which is available to authorized users.

## Background

There was a substantial increase in hantavirus infections in 2017. Although primarily affecting the kidneys, this case draws attention to the fact that hantavirus might also involve the myocardium resulting in hantavirus-induced myocarditis which can be adequately 1) detected, and 2) followed by the use of a comprehensive cardiovascular magnetic resonance (CMR) assessment since CMR has proven high diagnostic value for detection of various myocardial pathologies, including patients with suspected myocarditis, cardiac sarcoidosis and other forms of non-ischemic cardiomyopathies [1, 2]. To the best of our knowledge there are no clinical studies showing hantavirus infection causing myocardial disease.

## Case report

A 36-year-old white male patient suffering from headache and abdominal pain presented at our emergency department. Initial ECG showed a sinus rhythm (40/min.) with a single T-wave inversion in lead V2, and an incomplete right bundle branch block. Thrombocytes were decreased with 71.000/μl (normal range 150.000–450.000/μl), creatinine was elevated (2.0 mg/dl) with a maximum increase to 3.0 mg/dl (normal range 0.6–1.1 mg/dl) and massive proteinuria. C-reactive protein was also elevated: 8.6 mg/dl (normal < 0.5 mg/dl). Puumala virus IgG ELISA turned out to be positive, and specific antibodies (IgG and IgM) could be detected in the serum, and confirmed by immunoassay, also see Additional file 1. The patient was admitted to the nephrology department for supportive therapy.

Six days later, the patient reported chest pain and dyspnea. High sensitivity troponin I rose up to 0.32 μg/l (normal range below 0.04 μg/l) with an increase of the creatinkinase to 319 U/l (normal max. 190 U/l), no dynamic ECG changes could be observed. The patient was admitted to the chest pain unit. Echocardiography revealed a normal left ventricular function (65%) without regional wall motion abnormalities, no pericardial effusion or valve abnormalities. Since creatinine has normalized in the meantime, coronary artery disease was ruled out by coronary CT angiography.

CMR for work-up of suspected myocarditis was performed using a 1.5 T Magnetom Aera (Siemens Health Care, Germany). Cine-SSFPs revealed normal LV-EF (60%) with no wall motion abnormalities. A modified Look-Locker inversion recovery product sequence (MOLLI, MyoMaps) was used for T_1_-mapping and performed in a single mid-ventricular short-axis (SAX) slice at mid-diastole, prior and after application of contrast agent according to current recommendations [3]. T_2_-mapping was performed in the corresponding mid-ventricular SAX before administration of contrast agent using an ECG-triggered T_2_-prepared single-shot steady-state free precession (SSFP) product sequence with multiple T_2_ preparation times [4]. Normal values: native T1 < 1000 ms, T2 < 50 ms. Analyses were made by cvi42 software (Circle, Canada). Late gadolinium enhancement (LGE) images were acquired after contrast administration (Gadobutrol 0.15 mmol/kg) using segmented inversion-recovery fast low angle shot (IR-FLASH).

**Fig. 1:**
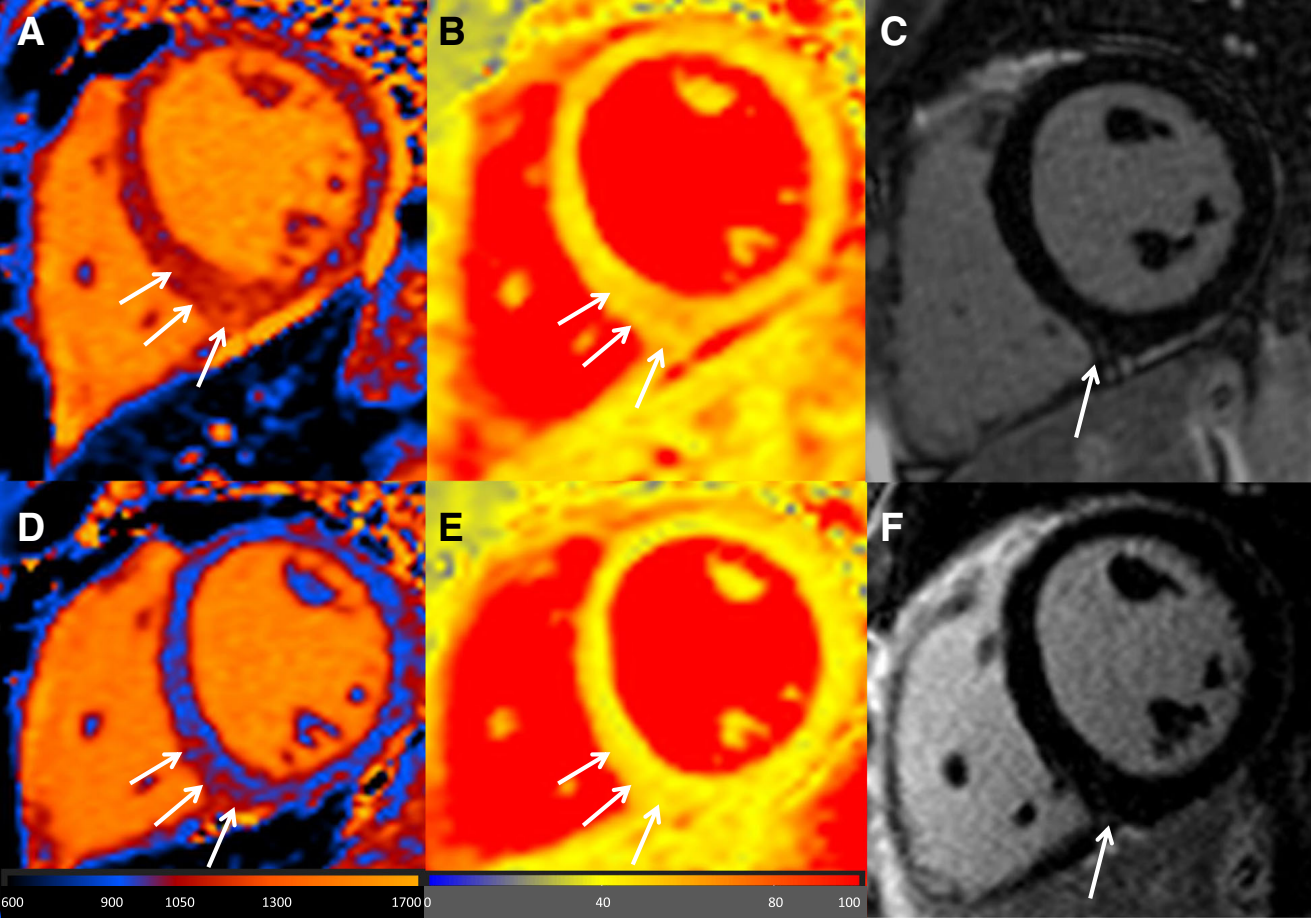
Representative mid-ventricular short axis slice from the presented patient displaying native T_1_-mapping technique (**a**), T_2_-mapping technique (**b**), and late gadolinium enhancement (LGE) images (**c**). The upper row visualizes the baseline exam. In contrast to the mapping techniques (**a** & **b**), which show increased values with preponderance in the inferoseptal wall in comparison to normal values (native T_1_: 1068 ± 73 ms in the entire slice vs. 1122 ± 31 ms in the inferoseptal wall; T_2_: entire slice 52 ± 6 ms, inferoseptal wall 55 ± 6 ms) no specific LGE could be detected in the inferoseptal wall (see arrows). In the bottom row, follow up images in the identical mid-ventricular short axis can be viewed. Note the signal decrease in the mapping images at follow up (**d** & **e**) especially in the inferoseptal wall (*arrows*) suggesting a dynamic process (native T_1_: 957 ± 58 ms in the entire slice vs. 971 ± 36 ms in the inferoseptal wall; T_2_: entire slice 44 ± 5 ms, inferoseptal wall 45 ± 3 ms), whereas the LGE image (**f**) turned out to be still negative

**Fig. 2:**
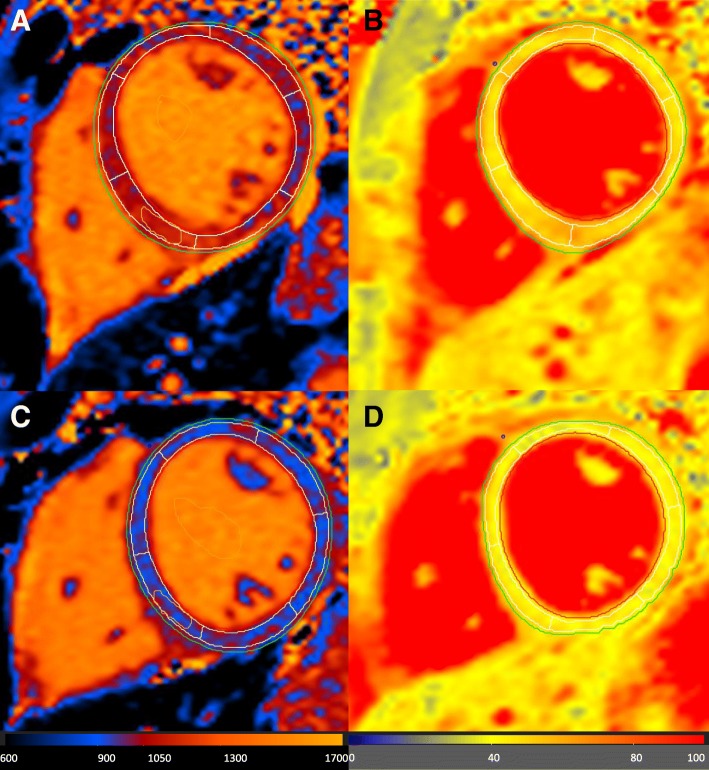
Demonstrate the analysis in detail of the native T_1−_ (**a**) and T_2_-mapping (**b**) images from baseline (upper row) and native T_1−_ (**c**) and T_2_-mapping (**d**) images at follow up (bottom row). Since increased native T_1_-mapping values can reflect both fibrosis and tissue edema, T_2_-mapping can discriminate between both stages by detecting myocardial water content (=edema/inflammation). In this case, increased native T_1_ and T_2_ values at baseline exam indicated substantial inflammation (myocarditis), which decreased at follow up suggesting myocardial healing which is supported by the absence of LGE (indicating irreversible myocardial damage) both at baseline and follow up CMR

Native T_1_-mapping demonstrated markedly elevated T_1_ values with preponderance in the inferoseptal wall (1068 ± 73 ms in the entire slice vs. 1122 ± 31 ms in the inferoseptal wall), also see Figs. 1a and 2a. Furthermore, T_2_-mapping revealed increased values (entire slice 52 ± 6 ms, inferoseptal wall 55 ± 6 ms), suggesting myocardial edema representing active myocardial inflammation by hantavirus infection. In contrast, the LGE image, potentially indicating irreversible myocardial damage if positive, in the corresponding slice was negative (Fig. 1c). Despite negative LGE, this patient was considered having hantavirus-induced myocarditis due to: 1) clinical symptoms, 2) increased cardiac biomarkers, 3) exclusion of CAD and 4) conspicuous native T_1_- and T_2_-mapping values detected by CMR.

In the next few weeks, the patient’s state of health rapidly improved and symptoms of chest pain and dyspnea disappeared. Five months later, the patient was followed up by the same CMR protocol: Substantial decrease of native T_1_ values (957 ± 58 ms in the entire slice vs. 971 ± 36 ms in the inferoseptal wall) and T_2_ values (entire slice 44 ± 5 ms, inferoseptal wall 45 ± 3 ms) in the mid-ventricular slice position could be observed, again LGE-negative, suggesting myocardial healing (Fig. 1 d-f).

## Discussion and conclusions

Hantavirus disease is one of the five most common notifiable viral diseases in Germany [5] with a rapid increase of infections in the year 2017 [6]. The viruses are RNA viruses of the Bunyaviridae family for which rodents are the natural reservoir. They can lead to haemorrhagic fever with renal syndrome in Asia and Europe, and hantavirus cardiopulmonary syndrome in America with reported case fatality rates of up to 35% [7].

Puumala virus is by far the most frequent cause of disease, which is characterized by acute kidney injury associated with thrombocytopenia and often proteinuria. Very few data is available about potential myocardial involvement [8, 9]. Although discrete ECG changes may occur [9], cardiac function determined by echocardiography might be preserved. Therefore, an adequate non-invasive diagnostic tool is warranted. Since CMR provides excellent tissue characterization, this technique is able to delineate potential myocarditis, which is known to have severe impact on the patients’ prognosis [10].

Myocardial inflammation leads to hyperemia with development of intracellular and interstitial edema, sometimes yielding irreversible myocyte damage, which can be visualized accurately by LGE. However, tissue edema without irreversible myocyte damage cannot be displayed by LGE. Furthermore, the value of LGE in displaying diffuse myocardial alterations is limited. Therefore, the sensitivity may be increased by combining LGE with recent T_1_- and T_2_-mapping CMR techniques capable of detecting reversible or diffuse myocardial injury. Since increased native T_1_ values might represent both fibrosis and edema/inflammation, T_2_-mapping by indicating myocardial water content (=edema/inflammation) is useful in discriminating between both stages. Therefore, it seems reasonable to combine LGE as well as T_1_-mapping and T_2_-mapping techniques performing a comprehensive CMR assessment [1, 2, 11].

Although endomyocardial biopsy is the gold standard for diagnosis in suspected myocarditis it is invasive and might suffer from the sampling error. ECG and echocardiography are ubiquitous available and still the method of choice for initial evaluation of potential myocardial damage but these lack specificity. Thus, a comprehensive CMR approach consisting of 1) recent T_1_- and T_2_-mapping techniques, which can detect also diffuse myocardial alterations by quantitative means in absolute values and can better separate between fibrosis and edema, and 2) established LGE, appear as the most reasonable non-invasive approach in patients with suspected myocarditis for both initial diagnosis and follow-up.

To the best of our knowledge, this is the first case demonstrating the benefit of a comprehensive CMR exam in a patient presenting with acute hantavirus-induced myocarditis. This case demonstrates that a CMR protocol including recent mapping techniques and established LGE is an adequate non-invasive tool for both 1) initial detection, and 2) follow up of patients with hantavirus-induced myocarditis, which might be more common than previously known.

## Additional file

Additional file 1: **Figure S1.** The recomLine HantaPlus IgG, IgM line immunoassay from Mikrogen (Neuried, Germany) detects Puumala Virus (PuN), Sin Nombre Virus (SinN), Hantaan Virus (HaN), Dobrava Virus (DobN) and Seoul Virus (SeoN). The assay detects the serotype specific N-terminal part of the virus nucleocapsid antigen. This test provided strong evidence for infection with Puumala Virus (IgG and IgM strong positive) in our patient. (PPTX 297 kb)

## Abbreviations

CAD: Coronary artery disease
CMR: Cardiac magnetic resonance
CT: Computed tomography
ECG: Electrocardiogram
ELISA: Enzyme-linked immunosorbent assay
Ig: Immunoglobulin
IR-FLASH: Inversion-recovery fast low angle shot
LGE: Late gadolinium enhancement
MOLLI: Modified Look-Locker inversion
SAX: Short axis
SSFP: Steady-state free precession
