# Supplementary figures and images for: Young patient with hantavirus-induced myocarditis detected by comprehensive cardiac magnetic resonance assessment
Source: BMC Infect Dis. 2019 Jan 6;19:15. doi: 10.1186/s12879-018-3658-8 (PMC6322348; doi:10.1186/s12879-018-3658-8)

## Slide 1
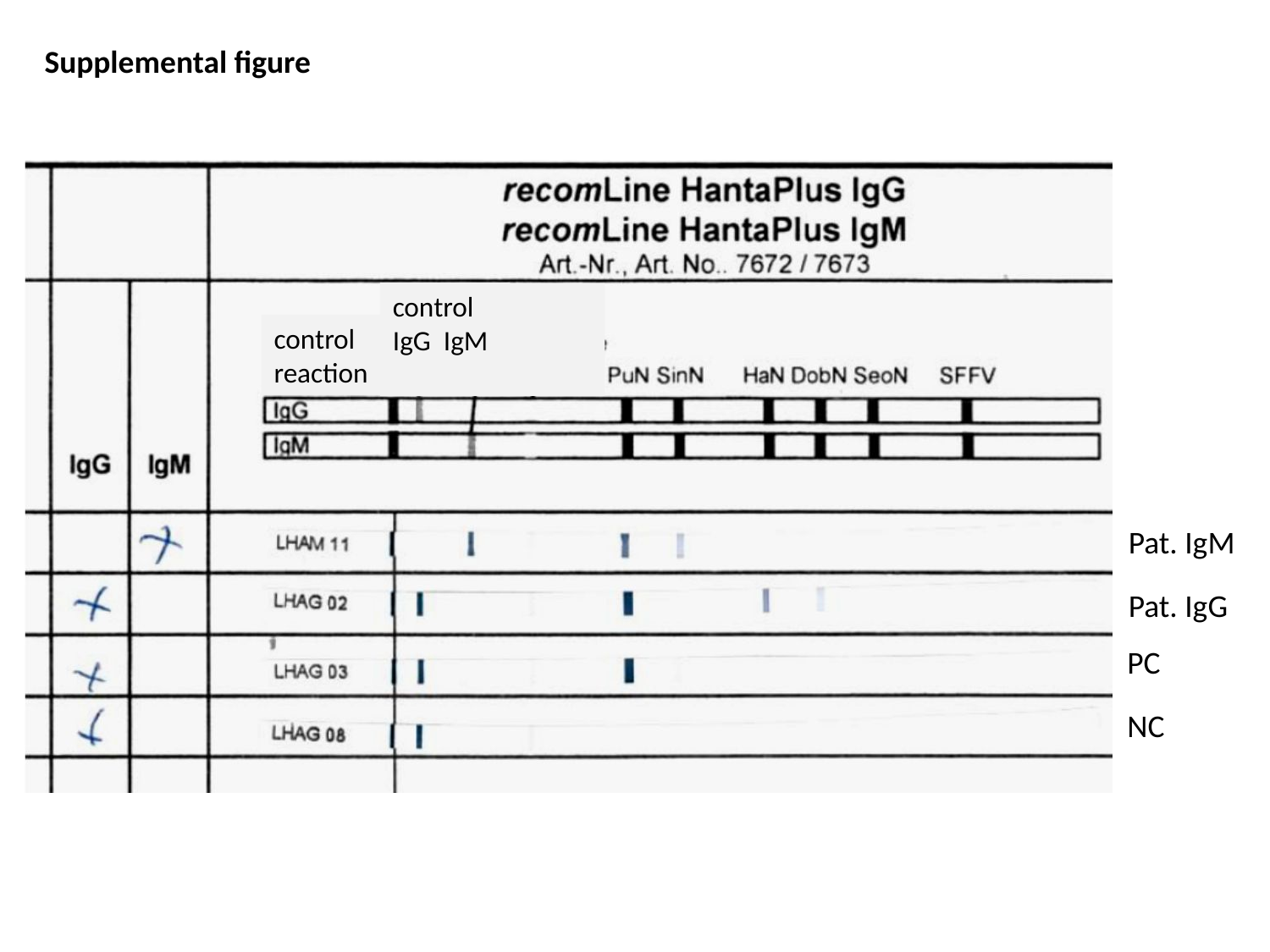

Supplemental figure
control
IgG IgM
control
reaction
Pat. IgM
Pat. IgG
PC
NC

Supplement: Supplementary file 1 — Figure S1. The recomLine HantaPlus IgG, IgM line immunoassay from Mikrogen (Neuried, Germany) detects Puumala Virus (PuN), Sin Nombre Virus (SinN), Hantaan Virus (HaN), Dobrava Virus (DobN) and Seoul Virus (SeoN). The assay detects the serotype specific N-terminal part of the virus nucleocapsid antigen. This test provided strong evidence for infection with Puumala Virus (IgG and IgM strong positive) in our patient. (PPTX 297 kb) [file 12879_2018_3658_MOESM1_ESM.pptx]
